# Supplementary material for: A high-resolution mRNA expression time course of embryonic development in zebrafish
Source: eLife. 2017 Nov 16;6:e30860. doi: 10.7554/eLife.30860 (PMC5690287; doi:10.7554/eLife.30860)
Supplement: Supplementary file 6. [file elife-30860-supp6.zip › biolayout-clusters-files/Cluster085-genes.html]

Cluster085


# Cluster085: Genes

| Ensembl ID | Gene Name | Chr | Start | End | Biotype |
| --- | --- | --- | --- | --- | --- |
| ENSDARG00000026329 | arhgap29a | 24 | 28364388 | 28478142 | protein\_coding |
| ENSDARG00000055128 | armc4 | 12 | 22905541 | 23027050 | protein\_coding |
| ENSDARG00000061848 | col15a1b | 2 | 24281303 | 24357609 | protein\_coding |
| ENSDARG00000045909 | dynlt1 | 4 | 372987 | 376994 | protein\_coding |
| ENSDARG00000026599 | eng2a | 7 | 40708851 | 40713383 | protein\_coding |
| ENSDARG00000004635 | epha7 | 20 | 24918746 | 25063979 | protein\_coding |
| ENSDARG00000034643 | fhl3a | 16 | 33701860 | 33782801 | protein\_coding |
| ENSDARG00000087196 | fibinb | 7 | 34018901 | 34020425 | protein\_coding |
| ENSDARG00000087897 | h2afy2 | 13 | 7420831 | 7435398 | protein\_coding |
| ENSDARG00000075325 | mrpl16 | 1 | 529209 | 535756 | protein\_coding |
| ENSDARG00000078725 | nos1apa | 6 | 35116514 | 35326476 | protein\_coding |
| ENSDARG00000103026 | p3h2 | 22 | 24362719 | 24455169 | protein\_coding |
| ENSDARG00000028148 | pax2a | 13 | 29640387 | 29674090 | protein\_coding |
| ENSDARG00000076799 | rnd3a | 22 | 12406831 | 12424795 | protein\_coding |
| ENSDARG00000077467 | sox10 | 3 | 2013057 | 2022138 | protein\_coding |
